# Peri-Traumatic Near-Infrared Light Treatment Attenuates the Severity of Noise-Induced Hearing Loss by Rescuing (Type I) Spiral Ganglion Neurons in Mice

**DOI:** 10.3390/brainsci15010062

**Published:** 2025-01-11

**Authors:** Max Meuser, Susanne Schwitzer, Parisa Faraji, Arne Ernst, Dietmar Basta

**Affiliations:** Department of Otolaryngology at Unfallkrankenhaus Berlin, Charité Medical School, University of Berlin, 12683 Berlin, Germany

**Keywords:** noise-induced hearing loss, near-infrared light, spiral ganglion neurons

## Abstract

Background: Previous studies have shown that multiple post-traumatic irradiations of the cochlea with near-infrared light (NIR) can significantly reduce noise-induced hearing loss. However, a single NIR pre-treatment was shown to have the same effect. Extending the pre-treatment time did not result in any further reduction in hearing loss. The present study investigated whether a combined NIR pre- and post-treatment had an increased effect on hearing preservation. Methods: Frequency-specific auditory brainstem potential thresholds (ABR) were determined in young adult mice. One group (n = 8) underwent NIR irradiation (808 nm, 120 mW, 15 min) of the cochlea, followed by a 30 min noise exposure (5–20 kHz, 115 dB SPL). A post-NIR treatment was administered for 30 min immediately following the noise trauma. After 14 days, hearing loss was determined by ABR measurements. The results were compared with a trauma-only group (n = 8) and an untreated control group (n = 5). Subsequently, the spiral ganglion neuron density was investigated. Results: A peri-traumatic NIR treatment resulted in a significantly lower hearing loss compared to the trauma-only group. Hearing protection in these animals significantly exceeded the effect of an exclusive pre- or post-treatment across all frequencies. A loss of spiral ganglion neurons in the trauma-only group was observed, which was significantly rescued by the peri-traumatic NIR treatment. Conclusions: A single peri-traumatic NIR treatment seems to be the more effective approach for the preservation of hearing thresholds after noise trauma compared to an isolated pre- or post-treatment. One target of the protective effect seems to be the spiral ganglion.

## 1. Introduction

As noise-induced hearing loss (NIHL) is the second most prevalent cause of hearing impairment after presbyacusis [1,2], its impact on individual patients and healthcare systems is considerable. NIHL is triggered by exposure to noise at work, in the environment, or during leisure activities and causes multifactorial damage to the auditory structures, e.g., mechanical damage of cochlear structures, oxidative stress, reduction in blood flow, and sterile inflammation, ultimately leading to the death of auditory sensory cells [3]. Consequently, animal experiments indicate that the loss of hair cells [4,5] and (type I) spiral ganglion neurons (SGNs) [6] are the main contributors to NIHL.

A plethora of pharmacological approaches have been pursued in an attempt to prevent noise-induced hearing loss. The most encouraging strategies identified to date include anti-inflammatory therapies, the utilization of antioxidants as suppressors of intracellular stress pathways, neurotrophic factors, the inhibition of programmed cell death pathways, and neurotransmission blockers [7]. The majority of these interventions are associated with unwanted side effects, such as the possible generation of cancer. A neuroprotective approach that has proven to be efficacious and is not associated with significant adverse effects involves the utilization of near-infrared (NIR) light. The application of NIR light in various medical disciplines has already been demonstrated [8]. Several animal studies showed a protective effect for noise-induced hearing loss mediated by NIR treatment after the noise trauma [9,10,11]. Experiments on rats conducted to study the effects of noise exposure have demonstrated a reduction in the loss of cochlear hair cells and a significantly lower hearing threshold shift of approximately 20 dB for a 12-day daily NIR treatment following trauma [12]. Interestingly, our group could show in mice that a single NIR pre-treatment (power density: 312 mW/cm^2^) of at least 10 min is sufficient to significantly reduce the hearing threshold shift after noise trauma in a similar amount [13]. Thus, a pre-treatment seems to be much more effective than a treatment applied after the trauma. However, extending the pre-treatment time up to 40 min did not result in a further reduction in hearing loss in these animal experiments.

The underlying physiological mechanism of NIR light effects is known as “photo-biomodulation” and is induced by light in the red to near-infrared range (630 to 1000 nm). Certain wavelengths within the NIR spectrum have been demonstrated to exert an influence on cytochrome c oxidase activity through the process of photon absorption. This, in turn, has been shown to result in a reduction in the efficacy of apoptotic mechanisms [14]. As a part of the mitochondrial respiratory chain, the cytochrome-c-oxidase constitutes the fourth component of the mitochondrial electron transport chain with absorption maxima of 680 and 830 nm, where cytochrome c is oxidized, and molecular oxygen is reduced to water [15,16]. In this process, one electron is transferred from each of four cytochrome c molecules to a single oxygen molecule, resulting in two molecules of water being produced. Concurrently, the four protons necessary for this process are transported across the mitochondrial membrane, resulting in the formation of a proton gradient that the ATP synthase enzyme utilizes to synthesize ATP. The activation of the cytochrome c oxidase can be attributed to the photodissociation of inhibitory nitric oxide (NO) [17]. As NO is non-covalently bound to the heme and Cu centers of the cytochrome c oxidase and competitively blocks oxygen, NIR irradiation enables the displacement of NO, facilitating a significant increase in respiration and, thereby, a notable elevation in ATP levels [14,18]. In vitro experiments have shown that the subsequent increase in cAMP levels, calcium release, and gene expression gives rise to a multitude of effects via downstream signaling cascades [19,20,21], unleashing the potential of NIR to prevent NIHL or mitigate its consequences.

As demonstrated by animal experiments, NIHL can lead to mitochondrial damage and dysfunction in the respiratory chain [22], resulting in a reduced production of ATP in cochlea cells [23,24,25]. Given that the production of endogenous antioxidants or the recovery of spiral ganglion cells following noise exposure is an energy-consuming process, transient ATP depletion may compromise the cochlea’s ability to defend against acoustic trauma [26]. Furthermore, noise-induced depletion of ATP activates the 5′ adenosine monophosphate- (AMP-) activated protein kinase (AMPK), which is the energy sensor protein that responds to fluctuations in the intracellular AMP/ATP ratio [27,28]. Concurrently with transient energy depletion, AMPK activates Rac-1, which, in turn, triggers the phosphorylation of the p67 protein [24,29]. P67 is one of the functional subunits of NADPH oxidase 3 (NOX3), which has been demonstrated to contribute to ROS overproduction [30]. In accordance with this, multiple authors describe an overload of ROS and reactive nitric species (NOS) in the cochlear after acoustic insult in guinea pigs and mice [31,32,33,34,35]. ROS and NO overwhelm the antioxidant enzyme system, including superoxide dismutase (SOD), catalase (CAT), glutathione peroxidase, and glutathione reductase [36,37,38]. The consequent imbalance of the antioxidant system can lead to an increase in apoptotic signals [38,39,40] and pro-inflammatory cytokines secretion [41,42], ultimately leading to cell degradation.

Considering the above-mentioned metabolic background of NIHL, an efficacious intervention to prevent NIHL appears to necessitate mainly anti-inflammatory, anti-oxidative, and anti-apoptotic effects. Furthermore, an effective method to enhance ATP generation in cochlear cells seems a prerequisite for a successful therapeutic approach.

Since animal experiments suggest that the effects of separate NIR pre-treatment or post-treatment seem to be saturated at approximately 20 dB hearing protection, a combined approach is possibly able to further enhance the therapeutic benefit of NIR treatment in the context of NIHL. Thus, the present study aims to ascertain whether a 30 min post-treatment in conjunction with a 15 min NIR pre-treatment has an amplifying effect on the protective efficacy of NIR on hearing thresholds in mice. Here, we further investigate the hypothesis that a protective effect of NIR could be mediated by a rescue of spiral ganglion neurons. We expect that the present study will provide further insights into the protective mechanisms of the NIR treatment on neuronal structures.

## 2. Materials and Methods

### 2.1. Animals

To avoid the influence of estrogen-related sex differences on the experimental outcomes, only female NMRI mice (Charles River Laboratories, Erkrath, Germany) were used. All animals were aged 10 to 12 weeks. Mice were housed in groups of four animals. Each cage was equipped with a resting house and enrichment features, including plastic tunnels and nesting material. Food and water were provided ad libitum, and the mice were kept in a 12/12 h dark/light cycle. Any kind of hearing impairment was defined as an exclusion criterion, and only animals with normal hearing were included in this study. Individuals with normal hearing were randomly assigned to one of the three experimental groups. These were the following groups: one group with noise exposure but without NIR treatment (trauma only; n = 8); one group without noise exposure or NIR treatment but with similar narcosis as applied for the other groups (sham control; n = 5); and one group with unilateral irradiation with NIR light for 15 min immediately before noise exposure and 30 min directly after the noise trauma (15-T-30; n = 8). The study protocol (approval number LAGeSo-G 0161/19) was approved by the Landesamt für Gesundheit und Soziales, Berlin, Germany.

### 2.2. Frequency-Specific Auditory Brainstem Response Measurements

Frequency-specific auditory brainstem responses (ABRs) were recorded for 5, 15, 25 and 35 kHz. ABR recordings were conducted under anesthesia (60 mg/kg ketamine, 6 mg/kg xylazine) immediately before and two weeks following a noise exposure. Subdermal needle electrodes were positioned on the mastoid (reference), vertex (active), and in one foot (ground). Tone stimuli were presented binaurally at varying sound pressure levels (SPL) using a sinusoid generator (FG 250 D, H-Tronic, Hirschau, Germany) and were calibrated with an audio amplifier (AMP-50, Tangent, Herning, Denmark). The brain stem responses were recorded using the MC-Rack system (USBME16-FAI-System, Multi-Channel Systems, Reutlingen, Germany). The loudness intensity was gradually reduced until the disappearance of wave II/IV indicated the ABR threshold level. Subsequently, the exact ABR threshold values were evaluated, with the experimenter being blind to the subject’s condition. The threshold shift for each frequency was calculated, and the hearing protective values were determined by subtracting the hearing threshold shift of the respective treatment group from the values of the corresponding trauma-only group. Results are presented as the mean hearing threshold shift in dB (±SD) for each experimental group.

### 2.3. NIR Treatment

**Figure 1:**
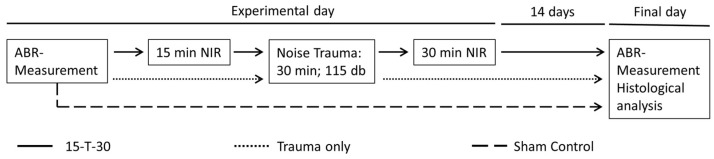
Experimental procedure for the experimental group (15 min of near-infrared light treatment (NIR) prior to noise trauma and 30 min of post-trauma treatment (15-T-30)), the trauma-only control (no treatment, only trauma (trauma only)), and sham control (no treatment or trauma).

The application of near-infrared light was facilitated by an adjustable isolated point laser module (DB808-120-3 (2265), Picotronic, Koblenz, Germany) emitting at a wavelength of 808 nm and a power of 120 mW. The laser module was placed at the outer ear canal at a defined angle and distance, enabling comprehensive coverage of the entire cochlea with the laser beam, which had an approximate diameter of 7 mm. The power density of the NIR light was 312 mW/cm^2^. A number of earlier studies have refuted claims of potential safety concerns regarding tissue damage, either in general or specifically in relation to the tympanic membrane, for the near-infrared (NIR) power density levels employed in the current study [43]. Two control groups were formed: one without noise exposure or NIR treatment but with similar narcosis as applied for the treatment group (sham control; n = 5) and one with noise exposure but without NIR treatment (trauma only; n = 8). Animals in the treated group (15-T-30; n = 8) were unilaterally irradiated with NIR light for 15 min immediately before noise exposure. The exposure time was selected based on a previous study from our laboratory, showing a significant effect of NIR pre-treatment on NIHL after 10 min but no relevant additional effect of longer treatment times [13]. A 30 min NIR post-treatment was carried out directly after the noise trauma (Figure 1). 

### 2.4. Noise Exposure

Each animal was subjected to a 30 min noise exposure within a soundproof chamber measuring 0.8 m × 0.8 m × 0.8 m, with a frequency-specific minimal sound attenuation of 60 dB. The noise exposure was conducted using a broadband white noise (5–20 kHz) at 115 dB SPL. The noise was delivered binaurally from a loudspeaker (HTC 11.19, Visaton, Haan, Germany) positioned above the animal’s head. The speaker was connected to an audio amplifier, providing the broadband noise from a DVD source (DK DVD-438, DK, Hamburg, Germany). The noise level was calibrated by utilizing a sound level meter (Voltcraft 329, Voltcraft, Hirschau, Germany) positioned in close proximity to the animal’s ear. Anesthesia was monitored via a video camera positioned within the illuminated chamber. Body temperature was maintained at a constant level of 37 °C using a heating pad.

### 2.5. Histological Analysis

Immediately after the last ABR recording, the tissue fixation was achieved by perfusion with 4% paraformaldehyde solution (PFA, pH 7.4, Sigma, Rödermark, Germany) via the left heart chamber. Inner ears were carefully removed from the skull and stored in 0.1% PFA at 4 °C for subsequent analysis.

The temporal bones, including the cochleae, were placed in 0.4 M EDTA for 14 days to ensure complete decalcification. EDTA solution was changed every second day. The cochleae were embedded in paraffin and sectioned frontal into 6 μm slices using a sliding microtome (Leica SM2000R, Leica Biosystems, Nussloch, Germany). The sections were stained using a modified haemalaun–eosin (HE) protocol. To deparaffinize the sections, slides were treated twice with Roti-Histol (Carl Roth, Karlsruhe, Germany) for 5 min each. The sections were rehydrated through a graded series of ethanol (90%, 70%) and distilled water, each for 5 min. Cellular nuclei were stained using haemalaun solution after Mayer for 5 min, followed by 10 min of differentiation under running tap water. The cytoplasm was stained with 0.1% eosin for 50 s, briefly rinsed in distilled water, and then differentiated in 70% ethanol. Dehydration was carried out through an ascending ethanol series (90% ethanol, isopropanol), followed by clearing in Roti-Histol. The slides were subsequently mounted using the Roti-Histokit (Carl Roth, Karlsruhe, Germany) for further analysis.

### 2.6. Image Analysis

**Figure 2:**
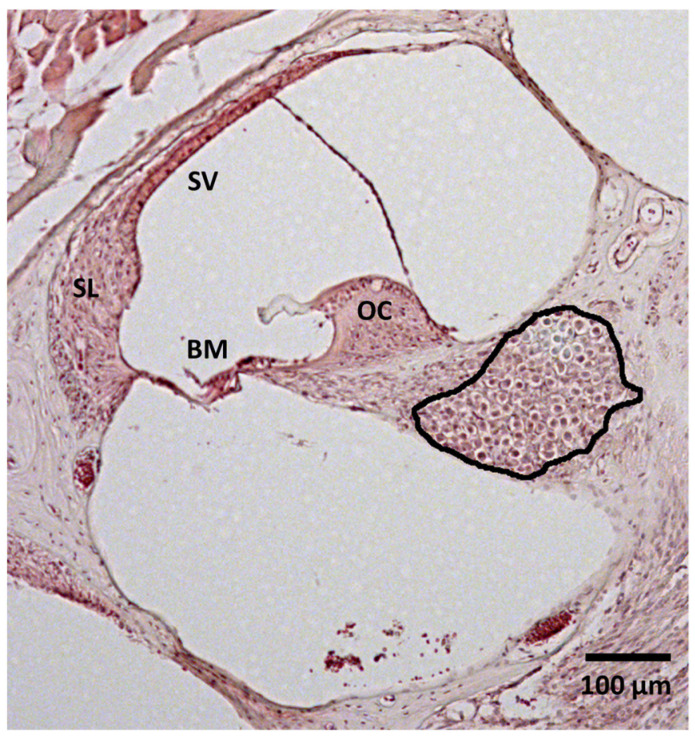
Histological analysis of the spiral ganglion neuron density. Exemplary image of a specimen from the 15-T-30 group. The Rosenthal canal is marked by the black line. OC = organ of corti; BM = basilar membrane; SL = spiral ligament; SV = stria vascularis.

The stained tissue sections were examined using a Zeiss AxioLab A1 microscope (Carl Zeiss, Oberkochen, Germany), and digital images of each Rosenthal canal (RC), including type I spiral ganglion neurons (SGNs), were captured with a Zeiss ICc1 camera (Carl Zeiss, Germany). Image analysis was performed using FIJI software (ImageJ version 1.53c, National Institutes of Health, Bethesda, MD, USA). First, each RC within the modiolus was outlined to determine its specific area (Figure 2). SGNs were counted using the cell counting tool of FIJI, and the SGN density was calculated. To ensure consistency in statistical comparisons, SGN densities were normalized to the mean RC area (mean of all analyzed RC on all sections included in the analysis) (mean area: 0.0244 mm^2^). At least 10 RCs were analyzed per animal. The results are presented as mean ± SD. To further investigate frequency-specific variations in SGN densities, the Rosenthal canal was analyzed separately in the basal (higher-frequency), medial, and apical (lower-frequency) regions.

### 2.7. Statistical Procedures

The data distribution of auditory threshold data and spiral ganglion neuron counts was evaluated using a Shapiro–Wilk test. Comparisons of frequency-specific auditory threshold data between the experimental and trauma groups were performed using the Mann–Whitney U test (for non-normally distributed data) or the *t*-test (for normally distributed data). Analysis of SGN density was conducted by a Kruskal–Wallis test, with a subsequent pairwise comparison using the Dunn–Bonferoni test, including a Bonferoni correction for non-normally distributed data or by a one way ANOVA, followed by a post-hoc Tuckey test for normally distributed data.

All statistical analyses were conducted using the SPSS software (IBM SPSS Statistics Version 25, IBM Corp., Armonk, NY, USA). The level of significance for all statistical tests was set at *p* < 0.05.

## 3. Results

### 3.1. ABR Threshold Shift

**Figure 3:**
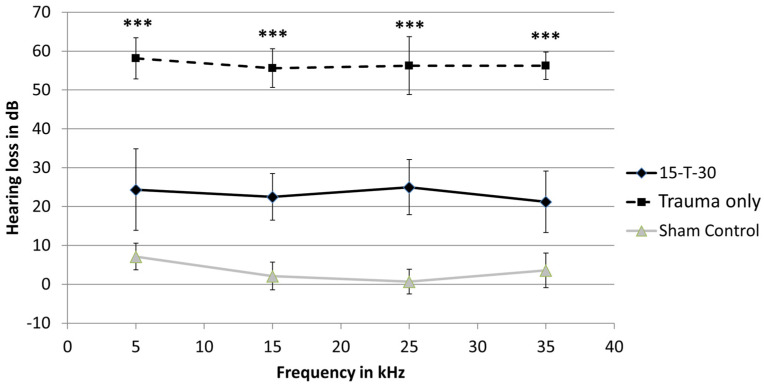
Noise-induced hearing threshold shifts. The mean (SD) of noise-induced hearing threshold shifts are shown for the 15-T-30, the trauma-only, and the sham control groups. Asterisks indicate the existence of significant differences between the 15-T-30 and the trauma-only group. *** = *p* ≤ 0.001.

The analysis of the ABR-determined hearing thresholds in the trauma-only group showed hearing threshold shifts of 58 dB (±5.3 dB) at 5 kHz, 55.6 dB (±4.9 dB) at 15 kHz, 56.2 dB (±7.4 dB) at 25 kHz, and 56.2 dB (±3.5 dB) at 35 kHz. In the 15-T-30 group, hearing threshold shifts of 24.3 dB (±10.50 dB) at 5 kHz, 22.5 dB (±5.9 dB) at 15 kHz, 25 dB (±7 dB) at 25 kHz, and 21.2 dB (±7.9 dB) at 35 kHz were observed. The analysis to determine whether the threshold shifts of the 15-T-30 group differed statistically from those of the trauma-only group was performed using a *t*-test for normally distributed data (at 15 and 35 kHz), and a Mann–Whitney U test was performed if the data were not normally distributed (at 5 and 25 kHz). The statistical analysis revealed a significantly lower shift in hearing thresholds in the 15-T-30 group in comparison to the trauma-only group at 5 kHz (U = 64; z = 3.4; *p* ≤ 0.001), 15 kHz (t(13.5) = −12, *p* ≤ 0.001), 25 kHz (U = 64; z = 3.3; *p* ≤ 0.001), and 35 kHz (t(9.6) = 11.4, *p* ≤ 0.001). The sham control group exhibited negligible hearing loss (Figure 3).

### 3.2. Hearing Protection

**Figure 4:**
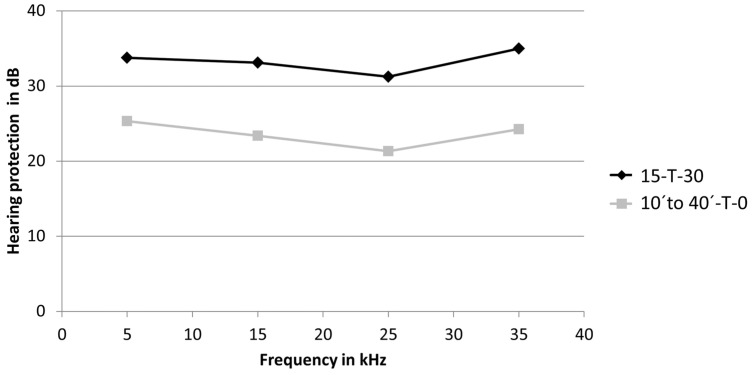
Hearing protection. The differences between the threshold shift of the treatment groups and the trauma-only group are shown for 15-T-30 and for the mean of the 10, 20, 30, and 40 min exclusive pre-treatment groups from [13] (10′ to 40′-T-0).

The calculation of the hearing protection as the difference between the threshold shift of the treatment group and the trauma-only group resulted in the protection of 33.7 dB at 5 kHz, 33.1 dB at 15 kHz, 31.2 dB at 25 kHz, and 35 dB at 35 kHz in the 15-T-30 group. The corresponding calculations of previously published treatment groups, which were subjected to an exclusive NIR pre-treatment with a duration of 10, 20, 30, and 40 min [13], demonstrated a mean protection capacity of these groups of 25.3 dB at 5 kHz, 23.3 at 15 kHz, 21.3 at 25 kHz, and 23.2 at 35 kHz. This comparison showed an increased protective effect in the 15-T-30 group of 8.4 dB at 5 kHz, 9.7 dB at 15 kHz, 9.9 dB at 25 kHz, and 10.7 dB at 35 kHz. Over the entire frequency range, the peri-traumatic NIR treatment resulted in a mean elevation of hearing protection of 9.7 dB compared to the exclusive pre-treatment groups (Figure 4).

### 3.3. Spiral Ganglion Neuron Density

For the analysis of statistically significant differences between the groups regarding the mean density of SGNs (±SD) normalized on the mean RC area (0.0244 mm^2^), the Kruskal–Wallis test was applied. As this test confirmed statistically significant differences between the experimental groups (H(2) = 60, *p* ≤ 0.001), a post-hoc pairwise comparison was performed using the Dunn–Bonferoni test incorporating a Bonferoni correction. 

**Figure 5:**
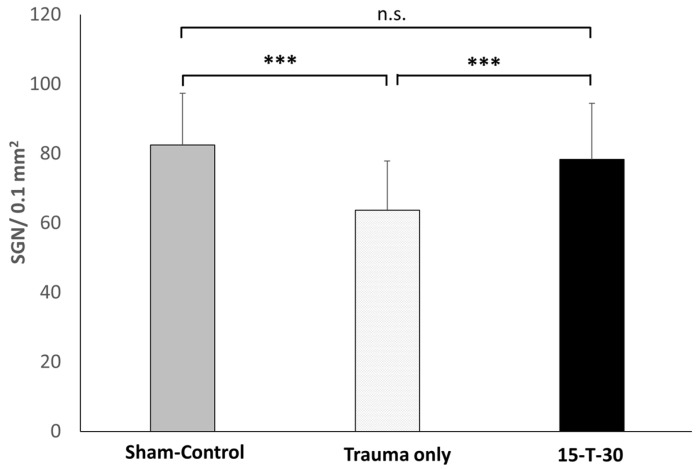
Spiral Ganglion neuron (SGN) density. Mean number of spiral ganglion neurons (±SD) in the sham control, the trauma-only, and the 15-T-30 groups. The SGN number was normalized by the average Rosenthal canal area of all samples. *** = *p* ≤ 0.001; n.s. = not significant.

In the sham control, which served as a Baseline for comparison, a mean of 82.4 (±14.8) SGNs was observed. In relation to this, noise exposure alone resulted in a statistically significant reduction in SGN counts in the trauma-only group, with a count of 63.7 (±14) (z = 7177; *p* ≤ 0.001) according to the Dunn–Bonferroni test. The mean SGN density for the 15-T-30 group was significantly higher (78.3 (±16.1)) compared to the trauma-only group (z = −5684; *p* ≤ 0.001). No significant difference was observed between the sham control and the 15-T-30 group (z = 1920; *p* = 0.164) (Figure 5).

In order to ascertain whether significant differences between the experimental groups in SGN density were present specifically in the basal, medial, or apical turn of the cochlea, a one-way ANOVA was initially conducted. This analysis revealed significant differences between the groups in the basal (F(2, 98) = 23.4, *p* ≤ 0.001) and medial (F(2, 85) = 11.8, *p* ≤ 0.001) turns, but not in the apical turn (F(2, 45) = 2.1, *p* = 0.0132). A Tukey HSD test was carried out post-hoc to ascertain which groups differed in the SGN density in the basal and/or medial turn. 

**Figure 6:**
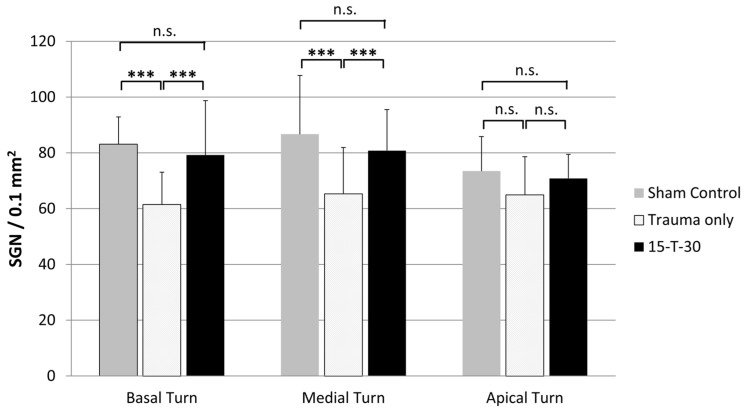
Comparison of the mean number of spiral ganglion neuron (SGN) density (±SD) in the basal, medial, and apical turns of the cochlea. Shown are the sham control, the trauma-only, and the 15-T-30 groups. The SGN number was normalized by the average Rosenthal canal area of all samples. *** = *p* ≤ 0.001; n.s. = not significant.

The separate analysis of the mean SGN density for the basal, medial, and apical turns revealed a mean number of 83.1 (±9.7) SGNs in the basal, 86.6 (±21.1) in the medial, and 73.4 (±12.3) in the apical turn of the sham control. In comparison with the trauma-only group (61.4 (±11.5) in the basal, 65.3 (±16.5) in the medial, and 64.9 (±13.6) in the apical turn), the Tukey HSD test revealed a significant difference in the basal (*p* ≤ 0.001; 95%-CI [13.5; 29.7]) and medial turns (*p* ≤ 0.001; 95%-CI [9.6; 33]) but not in the apical turn (*p* = 0.162; 95%-CI [−2.5; 19.6]). In the 15-T-30 group, the mean number of SGNs was 79.1 (±19.4) in the basal, 80.8 (±14.7) in the medial, and 70.8 (±8.5) in the apical turn. Compared to the trauma-only group, these cell numbers were significantly higher in the basal (*p* ≤ 0.001; 95%-CI [9.5; 25.8]) and medial (*p* ≤ 0.001; 95%-CI [5.6; 24.9]) turns but not in the apical turn (*p* = 0.342, 95%-CI [−4.2; 16]). There was no significant difference between the sham control and the 15-T-30 group in the basal, medial, or apical turns (Figure 6).

## 4. Discussion

The present study revealed a significant reduction in noise-induced hearing loss by applying a peri-traumatic NIR treatment. The mean protective effect, as calculated across the whole frequency range (5–35 kHz), was 33.3 dB. In comparison, a single exclusive pre-treatment with NIR achieved a protective effect of only 23.6 dB using the same experimental conditions [13]. Moreover, the application of an exclusive 12-fold NIR treatment after the noise trauma demonstrated a protective effect of 20 dB on average [12]. Thus, the peri-traumatic NIR treatment as applied in the present study was more effective as an isolated pre- or- post-treatment, even if the total treatment duration was much shorter compared to the isolated post-treatments (720 min compared to 45 min).

The peri-traumatic treatment carried out in this study demonstrates a further gain in the protective effect of approximately 10 dB in the case of a single pre-treatment and 13 dB in the case of an exclusive post-treatment. If these results could be repeated in humans, a reduction in hearing loss of about 10–13 dB would seem clinically relevant, as a 10 dB lower hearing threshold results, on average, in a 15% better speech understanding of monosyllabic words [44]. Moreover, it has been demonstrated that an incremental loss of hearing of 10 dB is associated with a notable decline in the outcomes of the physical, psychosocial, and overall Sickness Impact Profile in the context of presbycusis [45]. Additionally, Lin and Ferrucci (2012) have shown that a 10 dB increase in hearing loss is associated with a significant elevation of falls in a population aged between 40 and 70 years [46].

### 4.1. Mechanism of NIR Action in Peri-Traumatic Treatment Regime

Regarding the putative mechanism of action of the NIR treatment performed, the results of Bartos et al. (2016) show that a low dose of NIR (Fluence of 3 J/cm^2^) applied once before challenging the cells with oxidative stress leads to a reduction in superoxide radicals and NO in cultured hair cells [47]. Since superoxide radicals react with NO to form peroxynitrite, an inhibitor of cellular respiration [48], this could have resulted in an increase in intracellular ATP levels. However, the findings of Sharma et al. (2011) indicate a dose-dependent effect of NIR on the level of ROS and NO. The results demonstrated that fluencies between 0.03 and 3 J/cm^2^ resulted in an increase in ROS/NO levels in cortical neurons. In contrast, a fluence of 10 J/cm^2^ leads to a reduction in ROS/NO levels compared to 3 J/cm^2^. The highest tested fluence of 30 J/cm^2^, in turn, led to a further increase in ROS/NO levels compared to 10 J/cm^2^ [49]. In the present study, an irradiance applying a power density of 312 mW/cm^2^ was employed, which should result in a fluence of 280 J/cm^2^ with a pre-treatment time of 15 min and 560 J/cm^2^ with a post-treatment time of 30 min. Although the difference between our experiment on living animals and the in vitro experiments described in the literature limits the comparison, an increase in the ROS/NO level should be expected when applying these high fluencies. A possible protective mechanism on cochlea cells of NIR-induced increase in ROS levels was demonstrated in irradiated healthy normal embryonic fibroblasts. Here, irradiation activates cyclooxygenase and leads to an increase in the membrane potential, resulting in a short, moderate burst of ROS [29]. This activates NF-κB [50], which leads to protection against inducible nitric oxide synthase-triggered oxidative stress and caspase-3-mediated apoptosis [51]. In contrast, the NIR-induced increase in ATP appears to be independent of this mechanism. This is demonstrated by the results of a study conducted by Sharma et al. (2011) [49] on mouse primary cortical neurons. Here, the addition of antioxidant N-acetyl-cysteine to irradiated cells resulted in the blockade of NF-κB activation, but no change in mitochondrial membrane potential or increase in ATP was observed [50].

Furthermore, the oxidative status of the irradiated cells is an important factor in predicting an inhibitory or activating effect of NIR irradiation on ROS. Given that ROS is dose-dependently activated by NIR in non-stressed cells but inhibited in cells that have undergone oxidative stress [52,53], it can be postulated that the effect of the post-traumatic NIR treatment is based on the inhibition of ROS production following noise trauma.

The precise time point at which ROS will emerge is still under debate. The administration of noise trauma to guinea pigs resulted in an increase in O_2_^−^ as early as five minutes post-exposure in cochlea tissue, thus indicating that free radical accumulation is an early event in NIHL [31,32]. However, another study observed a delayed occurrence of nitrotyrosine and 4-HNE (histochemical markers of NO and ROS formation) with an onset at day seven after the acoustic insult [34]. This indicates a time window for therapeutic intervention after the noise trauma. Considering these two alternative scenarios, the impact of NIR post-treatment, as conducted in the present study without delay following noise trauma, may be either a direct inhibitory effect on immediate ROS production or the preparation of cells for the subsequent delayed increase in ROS.

### 4.2. Spiral Ganglion Neuron Density

The density of SGNs, the primary neurons of the peripheral auditory pathway, can decrease significantly as a consequence of noise trauma [6,54]. The analysis of SGN density in the present study demonstrated an average of 82.4 SGNs per mean RC area (0.0244 mm^2^) in the sham control, with a notable decline to 63.7 SGNs in the trauma-only group. These observations are in line with a recent study from our group showing 82.2 SGNs per mean RC area in the control group and 67 SGNs 7 days after the acoustic insult [6]. This comparison emphasizes that there is no recovery of SGN loss between the 7th and 14th day following the noise trauma. Our data suggest rather a mild progressive loss of SGNs over time. The peri-traumatic NIR treatment resulted in a significant rescue of 82% of the SGNs that were lost in the trauma-only group as a consequence of the acoustic insult. The discrepancy of the lack of SGN protection in the apical cochlear region should be related to the non-significant reduction in SGNs upon noise exposure in this area. The frequencies located tonotopically in this region are too low to be affected by the noise exposure (band pass 5–20 kHz). Also, the ABR measurements could not prove any effect on hearing thresholds in this area since the lowest test frequency was 5 kHz, which is not tonotopically located in the apical region.

We, therefore, suggest that the NIR-mediated rescue of SGNs reported here is an essential contributor to the shown hearing threshold protection in the context of noise trauma.

One criticism of the analysis is the sample size difference. The statistical power might be affected by having unequal sample sizes. In addition, the sample size itself is small. Larger studies should verify the present findings.

### 4.3. Potential for a Clinical Application

In order to adequately evaluate the clinical significance of the impact of NIR on NIHL demonstrated in this study, a comparison with existing treatment options, like antioxidants and anti-inflammatory and anti-apoptotic drugs, is pertinent. Although most of these approaches are still being investigated, and it remains to be seen which treatment method will become part of everyday clinical practice, the use of steroids for the treatment of NIHL has established itself as today’s standard therapy. Nonetheless, a recently published three-arm, multicenter, randomized, triple-blind study on the efficacy and safety of systemic high-dose glucocorticoid therapy and a related Cochrane review of intratympanic therapy of sudden hearing loss with Glucocorticoids show little to no effect. This applies to systemic and intratympanic therapy and the corresponding combination [55,56].

In this regard, NIHL treatment with NIR could be a promising candidate for clinical application. A further argument in favor of the clinical use of NIR therapy is the expected low cost when compared to available standard therapies. With the exception of the purchase of a suitable laser module, the cost is negligible. Furthermore, a positive side effect profile of NIR treatment without serious side effects, such as those that can be observed with steroid treatment (e.g., osteoporosis, osteonecrosis, and cataracts [57]), are not known for irradiation with near-infrared light. However, it should be noted that prolonged exposure to a high-power NIR can lead to an increase in temperature in the cochlea and, thus, to undesirable effects. The current limitations pertinent to the clinical usage of the NIR treatment include the challenge of determining and applying the optimal amount of radiation energy to the cochlea. Surprisingly, a recent clinical study [58] could not show a reduced hearing threshold shift upon an NIR treatment applied before a moderate noise exposure (94 dB for 15 min) compared to controls. A temporary threshold shift can be induced by such moderate noise exposure in humans with a specific vulnerability to noise. Thus, protective effects of an NIR pre-treatment upon moderate noise exposure would be expected in such individuals. Unfortunately, the sample size in this study was very small, and the noise-induced threshold shift varies a lot between the study participants. This suggests that most of them had no specific susceptibility to noise. A group effect was not achievable in this study since the group was too inhomogeneous in their reaction to noise. Furthermore, it is unclear if the NIR light reached the cochlea of the participants.

This points to a further challenge in terms of standardization; the laser must be precisely positioned at a distance and angle to the cochlea to enable a successful treatment, as shown in various animal studies (see overview in [10]). These challenges are the focus of an ongoing study. Furthermore, future studies should evaluate whether combination therapy of NIR with, e.g., steroids could provide an additional benefit for NIHL patients.

## 5. Conclusions

In mice, a peri-traumatic treatment with NIR was shown to have a significant additional protective effect on hearing thresholds compared to an exclusive pre- or post-treatment. This is functionally reflected by an impressive conservation of spiral ganglion neurons. Further evaluation is required to ascertain the transferability of these animal data to clinical applications in humans. 

## Data Availability

The raw data are available upon request due to legal (commercial) and ethical (animal research) reasons.
